# Resting Hormone Alterations and Injuries: Block vs. DUP Weight-Training among D-1 Track and Field Athletes

**DOI:** 10.3390/sports6010003

**Published:** 2018-01-16

**Authors:** Keith B. Painter, G. Gregory Haff, N. Travis Triplett, Charles Stuart, Guy Hornsby, Mike W. Ramsey, Caleb D. Bazyler, Michael H. Stone

**Affiliations:** 1 Center of Excellence for Sport Science and Coach Education, Sport, Exercise, Recreation and Kinesiology, East Tennessee State University, Johnson City, TN 37614, USA; keithbpainter@gmail.com (K.B.P.); STUARTC@mail.etsu.edu (C.S.); RAMSEYM@mail.etsu.edu (M.W.R.); bazyler@etsu.edu (C.D.B.); 2 Centre for Exercise, and Sport Sciences Research, Edith Cowan University, Joondalup 6027, Australia; g.haff@ecu.edu.au; 3 Health and Exercise Science, Appalachian State University, Boone, NC 28607, USA; triplttnt@appstate.edu; 4 Athletic Coaching Education, College of Physical Activity and Sport Sciences, West Virginia University, Morgantown, WV 26505, USA; william.hornsby@mail.wvu.edu

**Keywords:** strength, volume load, testosterone, cortisol, T:C ratio

## Abstract

Daily undulating periodization (DUP), using daily alterations in repetitions, has been advocated as a superior method of resistance training, while traditional forms of programming for periodization (Block) have been questioned. Nineteen Division I track and field athletes were assigned to either a 10-week Block or DUP training group. Year and event were controlled. Over the course of the study, there were four testing sessions, which were used to evaluate a variety of strength characteristics, including maximum isometric strength, rate of force development, and one repetition maximum (1RM). Although, performance trends favored the Block group for strength and rate of force development, no statistical differences were found between the two groups. However, different (*p* ≤ 0.05) estimated volumes of work (VL) and amounts of improvement per VL were found between groups. Based upon calculated training efficiency scores, these data indicate that a Block training model is more efficient in producing strength gains than a DUP model. Additionally, alterations in testosterone (T), cortisol (C) and the T:C ratio were measured. Although there were no statistically (*p* ≤ 0.05) different hormone alterations between groups, relationships between training variables and hormone concentrations including the T:C ratio, indicate that Block may be more efficacious in terms of fatigue management.

## 1. Introduction

The development of “periodized” resistance training programs has resulted in several programming models of training, each purporting to produce advances over more traditional models. “Daily undulating periodization” (DUP) programming models have been developed using daily changes in repetitions (and work) [1,2,3]. Heavy and light days are believed to be accomplished as a result of using loading differences (intensity) associated with the different repetition schemes being used from day to day. It is believed that this type of alteration produces enhanced variation such that superior gains in strength and power, and overall performance are accomplished [1,4].

Briefly, block periodization (Block) consists of stages, each containing three “blocks”: accumulation, transmutation and realization [5,6]. Typically, each block lasts 3–4 weeks. Conceptually, Block periodization depends upon “phase potentiation”, in which each individual block theoretically potentiates the next, through residual effects [5,6]. Generally, during accumulation, the focus is on higher volume and less specific training such that alterations in aspects, such as body composition, work capacity, and basic strength, are emphasized. Transmutation moves to more specific exercises with lower volumes and somewhat higher intensities of training, and can result in substantial increases in maximum strength for specific exercises. For strength-power athletes, realization typically deals with power oriented task-specific exercises and typically involves a taper (reduction in volume) to reduce accumulated fatigue. Often, a planned overreaching phase is used in conjunction with the taper [5,6,7].

A major difference between periodization models, particularly in most studies comparing Block and DUP, is the use of maximum repetition (RM) zones or consistent training to failure in both groups [3]. As reported by Painter et al. [8] and others, [7,9,10]. Block used constant repetitions during each block, but created heavy and light days by decreasing the load on light days, thus decreasing volume load (work) and intensity. Block also used percentages to adjust intensity and never trained to failure. Training to failure consistently (consistent relative maximum) may lead to greater fatigue and longer recovery time [5,6,11].

There have been few studies comparing the efficacy of these two programs, especially as it concerns fatigue management, hormonal alterations or incidence of injury among athletes. As part of the Painter et al. [8] study, absolute gains in maximum strength were not statistically different between Block and DUP models. However, the Block group showed superior effect size alterations and the efficiency of gain was markedly superior when compared to the DUP group. Efficiency in training is linked to the energy expended versus the performance outcome, as well as overall accumulated fatigue [11,12]. This would indicate (1) the amount of work is not the primary factor in altering performance, but rather how the work is manipulated and (2) that Block programming methods appear to manage fatigue in a superior manner. 

Training should be a process of optimizing stimuli and recovery-adaptation. This process requires providing a stimulus for adaptation as well as adequate management of accumulated fatigue [11,13]. Factors that can be affected by training variables include hormone alterations [14,15] and injury status [11,16]. Hormonal alterations that occur as a result of training are indicative of the athlete’s training state and are influenced by accumulated fatigue related to the stress of training [11,17,18,19]. Ideally, training variables, including fatigue, should be managed in a fashion such that hormone alterations can be used to optimize physiology and performance at critical points in the training process (e.g., to promote tissue hypertrophy and repair, and elevate performance as a result of a taper). 

Testosterone (T) and the catabolic stress hormone cortisol (C) are important indicators of strength-power performance and training strain. T and C likely produce both acute and chronic effects [20]. Acute hormonal effects deal with neural/psychological factors such as arousal and acute metabolic alterations such as the manner in which T augments cells ability to handle Ca^++^ during an exercise bout [21,22]. Chronic effects deal with several potential neural and metabolic alterations, particularly protein synthesis. Chronic effects, particularly with T, appear to operate in a “permissive” manner, such that positive adaptations can occur provided the hormone remains within reasonable physiological concentrations [11,20]. Permissive effects appear to be particularly important among advanced and elite athletes, as this may promote subtle adaptations [20]. Therefore, T and C can have substantial effects on psychological status, muscle physiology, strength, explosive strength, a function of the rate of force development (RFD), power, and markers of physiological development, performance and fatigue management [11,15,19,23]. Furthermore, resting T and C concentrations appear to be related to changes in resistance training volume and intensity [11,15,19].

Some data indicates that small alterations in T and C concentrations, even within physiological limits, may be related to meaningful changes in physiology, training strain and performance. Researchers have indicated that these hormone concentrations, and particularly the T:C ratio, can be a useful tool for monitoring training and tapering-induced performance alterations [11,17,18,19]. This ratio relates positively to body composition, maximum strength and general preparedness for sport [5,13]. Furthermore, programs that manipulate the T:C ratio appropriately may allow for enhanced athlete development in various sports, including individual sports (e.g., sprinting, throwing), and can aid in helping an athlete peak at a major competition [11,19]. Thus, potential alterations of endocrine function (e.g., T, C, T:C) may be an important aspect to consider in the development of training protocols. 

Recently a great deal of controversy has arisen over the relationship of training, particularly resistance training, volume and intensity, to injury risk. Evidence is mounting, indicating that sudden increases in volume or chronic large sport training intensities, and especially volumes, are associated with increased injury [24,25,26,27,28]. Strength training is generally considered to be a relatively safe process compared to other sports [29,30,31,32]. However, few longitudinal studies have investigated the relationship between resistance training volume and injury incidence, particularly in the holistic context of athletes in training. Indeed, based on these recent observations it is logical to believe that large volumes of resistance training, in addition to the athlete’s sport specific training, may increase an athlete’s susceptibility to injury. 

The aim of this study was to compare the effectiveness of the DUP programming method with one of a more traditional Block programming method for strength-power training, in a cohort of National Collegiate Athletic Association (NCAA) Division-1 track and field athletes, during a 10-week fall semester preparation-phase. A primary purpose of this study was to assess alterations in resting T, C, and the T:C ratio hormone levels; next (as part of a larger study [8]), outcome measures of strength (maximum isometric strength, instantaneous forces, rate of force development, 1RM parallel squat) and differences in volume load and training efficiency were collected, to compare the effectiveness of both training methods. Additionally, we assessed injuries across the 10-week training period, in relation to the previously-presented volume, intensity, and training efficiency data. 

## 2. Materials and Methods

### 2.1. Athletes

As part of an ongoing athlete monitoring program, twenty-three male collegiate level track athletes, 18–22 years of age, initially participated in this study. Prior to study initiation, sport medicine staff carried out health screenings that included a physical examination and questionnaires to ensure the athletes were free from injury or complaints when entering the study. Prior to study initiation, athletes were orally informed of the requirements for the study and potential risks, and then read and signed Institutional Review Board approved informed consent documents. Athletes primarily performing endurance activities (e.g., cross country) were excluded, due to different monitoring, scheduling and training requirements. Prior to initiation athletes were divided equally between groups, based on event, and year (freshmen or returner) [8]. Prior to study participation, athletes were deemed healthy and approved for practice by the sport medicine staff. If an athlete sustained any injury during the course of this study, the athletic training staff examined the athlete and documented the type and extent of the injury before return to training. For the study, data analysis exclusion criteria were missing three training sessions for any reason, missing any testing session, non-compliance with the prescribed protocol (e.g., lifting below the prescribed training loading schemes for 3 consecutive sessions) and performing any physical training outside of normal practice or strength training sessions. At the conclusion of the study, 4 athletes (1 from Block; 3 from DUP) had been excluded from the data analysis. Demographic data were, for Block (*n* = 10): height = 1.81 ± 0.10 m, age = 20.1 ± 1.3 years, mass = 93.2 ± 32.6 kg and % fat = 15.7 ± 11.6. Demographic data for DUP (*n* = 9) were as follows: height = 1.79 ± 0.47 m, age = 19.2 ± 0.7 years, mass = 86.0 ± 17.9 kg and % fat = 16.3 ± 7.5. There were no initial statistical differences in any characteristic.

### 2.2. Experimental Design

This study was part of an ongoing research and monitoring program, and used a randomized counter-balanced design, in which Division-I collegiate track and field athletes were divided into two resistance training groups, each performing either a traditional periodized programming model (Block) or a daily undulating training (DUP) programming model throughout the 10-week training period. Additional training (e.g., sprint, event practice etc.) was identical for each group. The training programs were constricted within the time limitations allotted by the athletic department and NCAA regulations. Performance variables and blood collection measurements were made at 0 weeks (T1), and after 4 weeks (T2), 8 weeks (T3) and 11 weeks (T4) after onset of the 10-week training period. 

### 2.3. Methodology

#### 2.3.1. Training

When comparing different training methods, it is essential to follow, as closely as possible, the guidelines of the methods. Both training programs (Block vs. DUP) were developed after an exhaustive literature review and were reviewed by multiple strength coaches, in order to assure that the program models were being applied appropriately. Exercises primarily consisted of squats, pulling movements and pressing movements, as these exercises and their programming are strongly related to the performances of these athletes [5,6,11]. For a more detailed description of training, see Painter et al. [8].

#### 2.3.2. Estimated Work

Estimates of resistance training work were based on volume load (repetitions × load × sets) [8]. At each session, coaches recorded the loads and repetitions completed for each set. Volume load and training intensity were then calculated using custom Excel spreadsheets [33]. Athletes were surveyed throughout the study, concerning additional exercise performed outside of normal training. Once each daily volume load was determined, each microcycle volume load was then summed. In order to calculate the training monotony, the average volume load per microcycle was determined and then divided by the microcycle’s standard deviation [34].

$$monotony\;\left(a.u.\right)=\frac{mean\;volume\;load}{standard\;deviation}$$

The amount of strain was then calculated by multiplying the monotony by the volume load.

(1) strain (a.u.)=monotony x volume load

Outside work, including practice, was regularly monitored by the researchers for accuracy and verified with the sport coaching staff. Coaches (*n* = 2) ranked each element of outside training (practice) on a subjective Likert-like scale, from 1–10 (1 = lowest and 10 = highest loading day). The coaches were asked to include their estimates of volume and intensity of the training in the rating producing coaching (subjective) assessment of the relative work performed by the athletes. As the groups were equated by event, both experimental groups performed essentially the same outside training. Each element of non-strength training was rated. These elements included sprinting, hurdling, karaoke, throwing, etc. The elements were summed to form an overall picture of relative training load (volume) for non-resistance exercise training. These data were used to ensure that both groups were performing approximately equal amounts of non-strength training work.

### 2.4. Testing

Due to training constraints, all testing occurred at the beginning of the week (Monday and Tuesday), on weeks one (T1), four (T2), eight (T3) and eleven (T4). All testing dates corresponded to the start of a new block of training for the Block group. Each athlete was familiarized with the testing and training protocols, on multiple occasions, several weeks prior to the T1 and the beginning of the program. All of the returners (non-freshman) had previously performed the tests prior to the familiarization phase, as they were part of an ongoing sport-monitoring program.

Testing consisted of hydration status, blood draws for resting T:C ratio, body composition, 1RM parallel squat and isometric mid-thigh pulls. A testing session took the place of the training session for that testing day and no other activity that day was permitted. Primary testing was conducted on Monday of each testing week (testing 1RM squats took place on Tuesday). All athletes were asked to fast for twelve hours before testing took place on day one to achieve optimal conditions for blood analysis, but were permitted to bring a snack (protein bar, etc.) to eat following the blood collection.

#### 2.4.1. Strength and Power Measures

The methodology for maximum strength evaluations has been reported in the first part of this study, “Strength gains: Block vs. DUP weight-training among track and field athletes” [8]. In brief, maximum strength was measured as isometric peak force (IPF), using an isometric mid-thigh pull and allometrically (N/(body mass (kg)^2/3^) scaled isometric peak force (IPFa). Isometric data was collected at 1000 Hz, using uniaxial force platforms (Rice Lake Weighing Systems, Rice Lake, WI, USA). Maximum dynamic strength was measured by evaluating the 1RM parallel squat (1RMSQ) performance. 

#### 2.4.2. Blood Collection/Testosterone and Cortisol

After checking hydration status, blood was collected from an antecubital vein, by certified personnel, using 21Gx 1–1/2” multiple sample needles and 8.5 mL (16 × 100 mm) clot activator blood collection tubes. All samples collected were stored on ice, allowing the blood to clot. After clotting, samples were centrifuged to separate the serum, and the serum was then transferred into smaller centrifuge tubes and frozen in a −80 °C freezer for later analysis. Samples were analyzed in one data set at the end of the study. Total testosterone (T) and cortisol (C) were measured by ELISA (DRG International, Mountainside); the intra assay Coefficient of Variation (CV) was 3.8% for T and 4.5% for C. The testosterone:cortisol ratio (T:C) was calculated as:

*T*:*C ratio* = *T*/*C* × 100 (2)

### 2.5. Monitoring Injuries

Although not a primary purpose of the study, injury may relate to the type of training protocol and fatigue management. Injury surveillance was supervised by the East Tennessee State University Sport Medicine Department. Injuries were evaluated and recorded by athletic trainers (National Athletic Training Association certified) and later analyzed to determine injury incidences. Athlete training time was modified depending upon the severity of injury, as evaluated by the sport medicine staff.

### 2.6. Statistical Analyses

Data were analyzed with SPSS (version 16.0; SPSS, Inc. Chicago, IL, USA). Multiple 2 × 4 repeated measure analysis of variance (ANOVA) were used to determine if statistical differences existed between the training interventions and the measurement times for all tested variables. A 2 × 10 ANOVA was utilized in the analysis of the volume load for each of the 10 weeks of training. Follow-up one-way ANOVAs were performed, to determine where statistical differences existed. Correlations were calculated using Pearson’s r (statistical significance was *p* ≤ 0.05, critical corrected r was total = 0.51, Block = 0.60, DUP = 0.66). The magnitude of r values were determined to be trivial 0.0–0.1; small 0.1–0.3; moderate 0.3–0.5; large 0.5–0.7; very large 0.7–0.9 or near perfect 0.9–1.0. Effect sizes (Cohen’s d using pooled standard deviations) were calculated. Effect sizes with CIs were assessed using the following scale: trivial 0.0–0.2; small 0.2–0.6; moderate 0.6–1.2; large 1.2–2.0; very large 2.0–4.0 [35].

## 3. Results

### 3.1. Maximum Strength

Alterations in maximum strength were determined and followed the same general pattern as previously noted in a larger group [8]. For example, maximum isometric force during the mid-thigh pull (IMTP) improved over time. Although there were no statistical differences between groups, the Block group generally showed greater percent gains and larger effect sizes in absolute and allometrically scaled maximum isometric strength characteristics over time. Reliability for all strength related measures were ICC ≥ 0.91. Over 10 weeks, IPFa increased in both groups. The Block group increased from 255.3 ± 46.2 to 291.3 ± 37.3 (% = 14.1, *p* = 0.06, ES = 0.90, CI = −0.05 to 1.85), and the DUP group increased from 229.5 ± 35.6 to 258.5 ± 33.8 (% = 12.6, *p* = 0.10, ES = 0.83, CI = −0.20 to 1.86). 

### 3.2. Training Volume Load, Intensity and Efficiency

**Figure 1:**
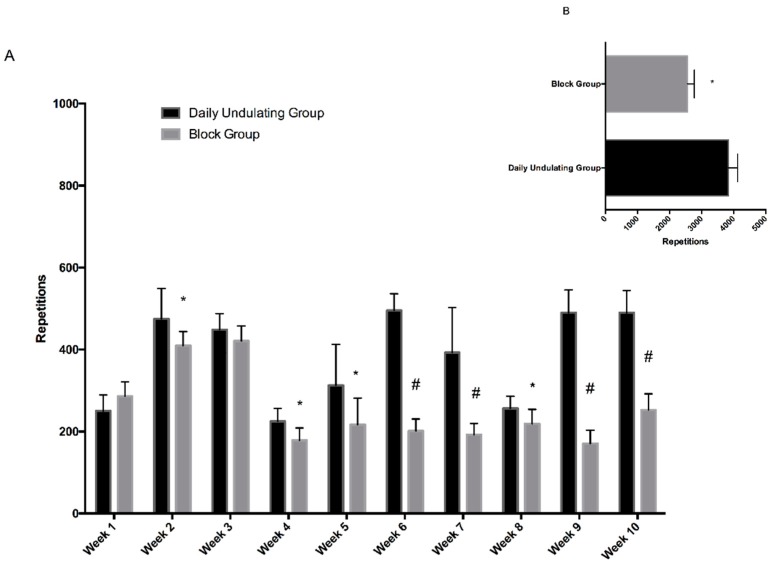
(**A**) Depicts repetitions completed each week in the daily undulating periodization (DUP) and Block groups; (**B**) depicts the mean repetitions completed by each group over the 10 weeks of training. Significant difference between groups: * *p* < 0.05, # *p* < 0.001. Daily undulating and block periodization repetitions.

As noted in our previous paper [8], the DUP group performed a statistically greater amount of work, compared to the Block group, to produce equal strength gains (strength data reported previously). Repetitions and estimated volumes of work (VL) were calculated weekly and for the duration of this study. The DUP group performed (*p* < 0.001, ES = 5.09, 95% CI = 3.07 to 6.65) more repetitions (+1274.2, +49.8%) than the Block group (Figure 1).

**Figure 2:**
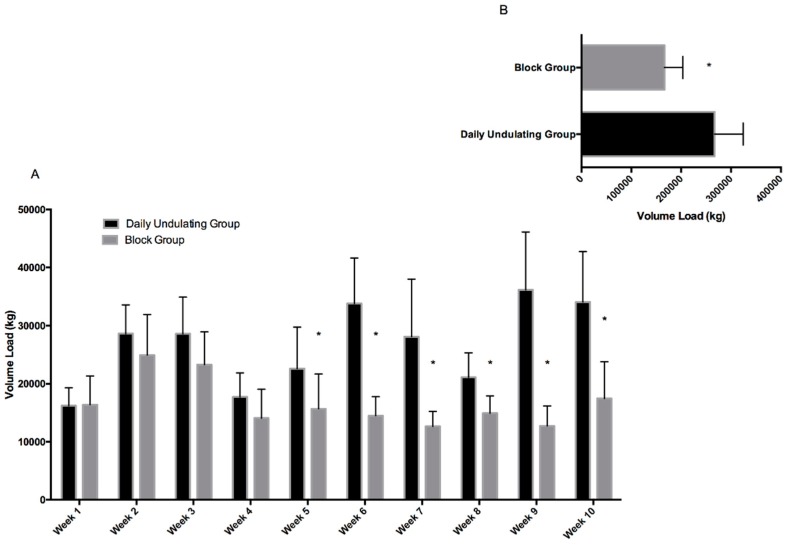
(**A**) Depicts volume-load completed each week in the DUP and Block groups; (**B**) depicts the mean volume-load completed by each group over the 10 weeks of training. Significant difference between groups: * *p* < 0.05. Daily undulating and block periodization volume load.

Additionally, the DUP group performed statistically (*p* < 0.001, ES = 2.11, 95%, CI = 0.91 to 3.13) more work by volume load (+100,455.8 kg, +60.3%) (Figure 2) than the Block training group.

There were no significant statistical differences (*p* > 0.05) between the DUP and Block groups for weekly training intensity or overall average training intensity (DUP = 70.4 ± 11.1 kg; Block = 66.5 ± 9.8 kg; *p* = 0.43, ES = 0.50, 95%, CI = −0.44 to 1.40). However, based on performance gains per unit of volume load and repetitions, a large statistical difference in the training efficiency between the two groups was noted.

### 3.3. Monotony and Strain

**Figure 3:**
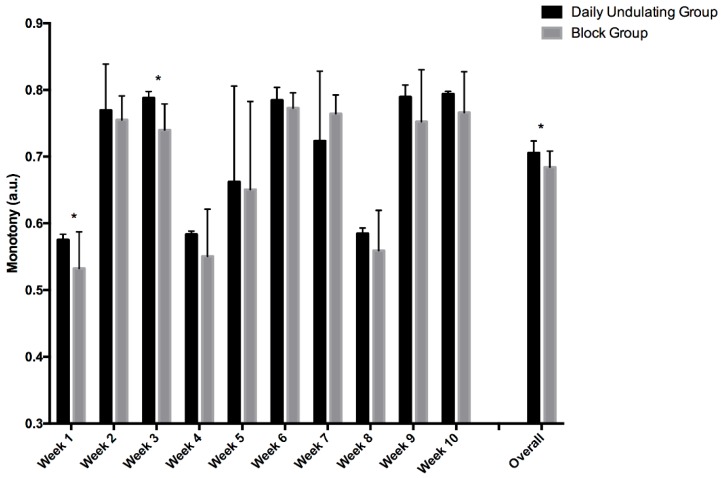
Depicts weekly training monotony scores in the DUP and Block groups. “Overall” represents the mean training monotony scores for each group over the 10 weeks of training. Significant difference between groups: * *p* < 0.05. Daily undulating and block periodization monotony scores.

**Figure 4:**
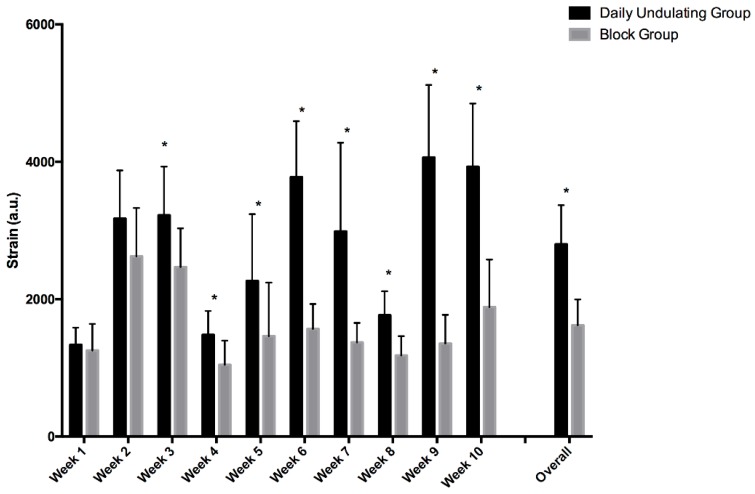
Depicts weekly training strain scores in the DUP and Block groups. “Overall” represents the mean training strain scores for each group over the 10 weeks of training. Significant difference between groups: * *p* < 0.05. Daily undulating and block periodization strain scores.

When comparing the DUP and Block groups, it was determined that the DUP group exhibited statistically higher average monotony (*p* = 0.037, ES = 0.98, 95%, CI = 0.01 to 1.87) and strain (*p* < 0.001, ES = 2.50, 95%, CI = 1.24 to 3.54) scores (Figure 3 and Figure 4). Specifically, the DUP group exhibited higher monotony scores (Figure 3) during week 1 (*p* = 0.027) and week 3 (*p* = 0.002). With the exception of weeks 1 and 2 (*p* = 0.76), the DUP group exhibited statistically higher strain scores (Figure 4) for all other weeks during the present study when compared to the Block group.

**Figure 5:**
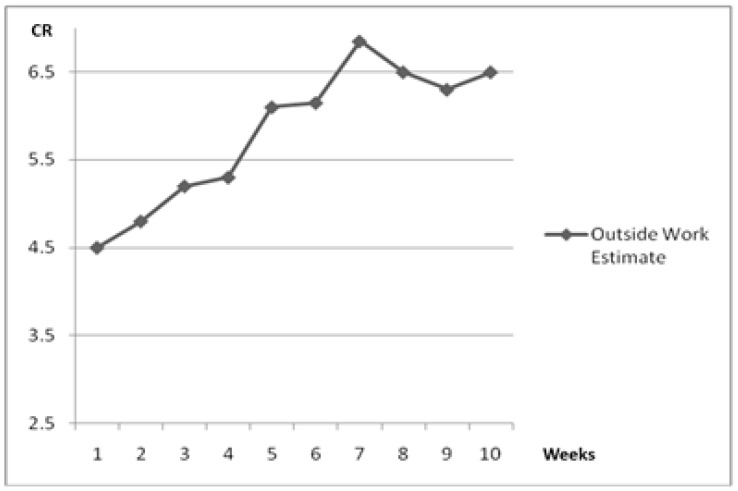
Outside work estimate. CR, coaches ranking of workload.

The estimated volume of outside work from the coaches’ rankings is shown in Figure 5. Generally, the amount of outside work increased through the first seven weeks, then showed a decrease.

### 3.4. Testosterone and Cortisol

The concentrations for T and C and the T:C ratio are shown in Table 1.

No statistically significant differences were found between groups for resting serum T and C concentrations. In addition, resting T and C concentrations did not statistically change from T1 through T4. In the combined groups, the initial T concentration correlated well with the final concentration (r = 0.51); C did not show this relationship (r = −0.16 ns); For Block, the correlations were r = 0.84 for T and r = 0.012 (ns) for C, and for DUP they were r = 0.27 (ns) for T and r = −0.67 for C. Correlations between % IPF and IPFa gain scores, and T and C, are shown in Table 2. 

Initial and final combined group T concentrations showed non-statistically significant weak to moderate relationships with % gain scores. The correlations for the initial T concentrations in the Block and DUP groups were similar to the combined group. Interestingly, the correlations for combined groups showed that initial and final C concentrations had strong negative correlations with % gain scores. However, the initial correlations for the Block group become stronger and more negative over time, but weaker for the DUP group. Thus, the groups showed an inverse pattern of correlation for C (Table 2). The correlations between the T:C ratio and percent gain are shown in Table 2.

Initial combined T:C ratios correlated strongly with IPF and IPFa. However, initial T:C ratios also correlated well with the change from T1 to T4, for both IPF and IPFa, for the Block group, but not in the DUP group. Additionally, the combined group T:C ratio at T4 had strong correlations with the % gain, in both IPF and IPFa, from T1 to T4, for the combined groups. This correlation (T4 vs. % gain) was somewhat higher in the Block compared to the DUP group. A very similar pattern of correlations was noted for the 1RMSQ % gain scores. 

### 3.5. Injuries

Based on sports medicine department records, there was a 40% injury incidence in the Block group, and a somewhat surprising 100% injury incidence in the DUP group. Injury severity ranged from minor cuts and abrasions to torn muscles and a back injury; however, no injuries were reported acutely during lifting sessions. Two athletes in the DUP had to be removed from the study as a result of injury during non-resistance training practice (back injury and hamstring pull); none were removed from the Block group. It should be noted that it is impossible to determine the exact cause of the injuries (i.e., practice versus weight-room or a combination). 

## 4. Discussion

This study indicates that, over a 10-week period, neither training protocol resulted in statistically significant alterations in hormone concentrations. However, the data do indicate that initial and final T, C and especially T:C ratios correlate with both initial maximum strength levels and improvements in maximum strength. Indeed, the T:C showed quite strong positive correlations with gains in maximum strength. These correlations may offer insight into the relative degree of fatigue management for Block and DUP training protocols. 

Testosterone has strong anabolic/anticatabolic agent properties [11,17,20]. Furthermore, T is also involved in growth and development of the nervous system [11,17,20]. As a result of these properties, T is strongly associated with a variety of performance-related characteristics, including muscle mass and lean body mass, maximum strength, RFD and power output [11,17,20]. Investigations of acute and short-term T alterations to resistance training have produced mixed results [11]. Some studies have observed increases in boys [36] and men [37] while others have demonstrated no difference or a decrease in T concentrations, even over relatively long periods of time [11,15,17,38]. When considering previous studies, large differences in training ages, biological ages, training states, level of athletes and types of resistance training programs implemented (volume, intensity etc.) may help explain the conflicting results. However, only a few studies have used athletes as subjects [8,15,17]. Crewther et al. [20] indicate that, among advanced and elite athletes, over relative long periods, little difference in training T concentration is typically observed, even though physiology and performance is altered. This observation is consistent with the concept that T may have a permissive effect on alterations in performance, including gains in maximum strength. In the present observation, this suggestion is consistent with the lack of statistically significant changes in T, even though there were substantial gains in maximum strength, particularly in the Block group.

Resting cortisol accumulation in the blood can result from altered metabolism, immunity and physical and emotional stress; thus, greater training strain coupled with outside stressors can result in greater concentrations. When first introduced to resistance training or increased training volume, human resting C concentrations tend to increase over several weeks. Subsequently, with continued training, C concentrations normally return to baseline or lower [11,18]. Furthermore, when training volume and/or intensity are lowered to more easily tolerated levels, C concentrations decrease, potentially enhancing the recovery-adaptation process and preparedness [11,19,39]. In the present study, based on percent alterations and effect sizes, both groups showed a decline in C at T4, compared to T1. However, the percent decline was approximately three times greater in the Block group compared to the DUP group (−19.2%; d = 0.56 vs. −6.5%, d = 0.21), again suggesting better fatigue management.

Testosterone and cortisol play important roles in neuromuscular development, and the ratio of these hormones (T:C) appears to provide a reasonable estimate of an athlete’s “anabolic environment”. The T:C ratio is related to fatigue management, overtraining potential and a number of physiological variables that can impact performance. For example, the T:C has been associated with lean body mass (LBM), leanness, strength and explosiveness [11,40,41]. Testosterone may have dual effects: long-term and short-term. Typically, the higher the resting T:C ratio, the greater the potential an athlete has to both develop strength and power (long-term effects) as well as acutely express strength-power characteristics (short-term effects) [11,19,20,23]. This ratio may reflect alterations in these variables more precisely than the concentrations of T or C alone [11,19,42,43]. As with the individual hormones, there is evidence that, among athletes, alterations in the volume of training can alter the T:C ratio, and that even minor alterations may be meaningful in terms of accumulative fatigue and performance [11,17,43]. The T:C ratio has been shown to be a viable marker of preparedness, thus altering the T:C ratio covaries with an athlete’s preparedness [11,15,19,23]. Periods of prolonged high-volume training can result in negative effects on the neuroendocrine system and a decrease in the T:C ratio. A chronic decrease in the T:C ratio can be associated with decreases in preparedness, and may be associated with an increased overtraining potential. However, a temporary elevation in training volume, or a period of planned overreaching or concentrated loading may help stimulate tissue growth and performance capabilities as a result of a rebound effect after returning to normal training [9,44]. However, continually training at high volumes can lead to prolonged increases in C and decreases in T, chronically lowering the T:C ratio, and potentially blunting muscle adaptations. A training stimulus that is too high in volume, for a long duration, will result in unmanageable accumulated fatigue, maladaptations, a decrease in the T/C ratio, increased injury potential and ultimately exhaustion (overtrained).

Maresh et al. [18] demonstrated that periodically measuring resting plasma concentrations of T and C in collegiate swimmers may be a useful method for monitoring physiological/performance changes that correspond to changes in the training program. Mujika and colleagues [19,45] suggest that very small differences in T and T:C can have considerable practical significance. They found a statistically significant correlation between the percent change in the T:C ratio and the percent change in increased swimming performance during a taper lasting four weeks in duration [45]. Specifically, regarding strength-power athletes, two studies [46,47] found increases in T:C during a 1 to 3 week taper, but these results were not statistically significant; both studies, however, demonstrated increases in performance.

In the present study, consistent with the literature [20], no statistically significant T:C alterations occurred across time. However, there are several interesting observations to consider that again may have practical significance. Initially the T:C ratios were higher in the DUP group; the ratio was inverted at T4, largely as a result of changes in resting C. Cortisol (C) is known to respond to a number of stressors, including alterations in work and training strain [20,48]. There was an increase in C for both groups, from T2–T3 (Block: % = 6.0, ES = 0.17; DUP: % = 11.5 ES = 0.68), This alteration in C (T2–T3) appears to coincide with the increase in outside work and may have been further affected by the marked differences in resistance training work (VL) during that period, as the DUP showed a larger increase.

During periods of reduced volume, the T:C can rise, coinciding with reductions in training volume [19]. There was a larger rise from T3–T4 in the Block group (74% d = 1.73) compared to the DUP group (50% d = 0.52) in response to the decreased volume load of resistance training and likely outside work. It should be noted that in the Block group, both the resistance and non-resistance training work decreased from T3–T4, whereas in the DUP group, only the outside work showed a decrease. Thus the total decrease in training volume was larger in the Block group, and this corresponded to a greater strength gain from T3–T4 in the Block group (See Table 2, Painter et al. [8]) as well as the larger increase in T:C. This indicates that tracking outside work (e.g., practice, sprints etc.) as well as estimates from resistance training may be valuable in future studies. Interestingly, the correlations between the T:C and performance values were consistently higher in the Block group and the pattern of correlations for hormones from T1–T4 suggests a consistently, and perhaps progressively more favorable, internal milieu for the Block group. While these alterations are quite subtle, they may indicate a better level of fatigue management in Block training compared to DUP training, and may relate to the greater efficiency of Block training [8]. Furthermore, these observed hormonal alterations may have played a role in the adaptations in strength characteristics; a role supported by the relationships found between strength characteristics and hormone alterations (Table 2). Although not out of the normal physiological ranges, it does appear that T and C can respond to temporary increases (concentrated load) or decreases (taper) in training volume [11,19]. Thus, the management of training variables and fatigue, possibly by monitoring the resting state T:C ratio, may be necessary to optimize performance and to avoid overtraining.

Consistent with the literature [31,32], no injuries occurred in the weight room. However, it is interesting that 100% of the DUP group sustained some form of injury, and two of these resulted in missed training and study drop out. From a programming perspective, it has been suggested that when training monotony and strain are both high, there is a higher risk for injury and illness [34,49]. In the present study, the DUP group demonstrated an overall higher monotony score that was coupled with a higher strain score. One of the tenants of the DUP model is the increased variation in training, but when examined on a microcycle level, this model of training results in a more monotonous training stimulus. Additionally, because of the loading parameters associated with this model, a higher training strain score is also noted, which, when coupled with the monotony score, increases overall injury risk. The data from the present study suggest that the DUP programming model demonstrates poorer fatigue management and an increased risk of overtraining and/or injury risk for athletes [50]. 

Thus, we argue that based on the hormonal alterations and total injuries over 10 weeks, the Block training model appears to manage fatigue in a superior manner. Superior fatigue management can markedly increase the fitness and preparedness of the athlete and raise the potential for enhanced performance, whilst probably also reducing the risk of injury. The present study was only 10 weeks in duration. Based on the data and observation of the athletes the authors believe that it is likely that longer-term programs would magnify the fatigue management issues associated with the DUP training model. This hypothesis is partially supported by the increasing efficiency score previously noted by Painter et al. [8]. Potentially, continual use of the DUP model among athletes, could result in a progression into a non-functional overreaching or overtraining state which would result in reduced performance capacity and an increased injury risk.

## 5. Conclusions

Fatigue management is an extremely important facet of training. Superior fatigue management can be associated with enhanced adaptation to training, increased performance, fewer injuries and general well-being of the athlete. For this study, we suggest that the Block programming approach can allow superior fatigue management and should be considered superior to DUP training because:

1. Training efficiency was greater and monotony and strain were lower.
2. Relationships between the T:C and maximum strength measures were consistently stronger.
3. The difference in the injury rate was quite large and may have resulted from superior fatigue management.

Based on this data we would suggest that this form of DUP type programming is a relatively inefficient method of resistance training, which may result in poor fatigue management and an increased risk of overtraining and injury potential when performed in conjunction with sport training. 

## Figures and Tables

**Table 1:** Resting Testosterone (T) and Cortisol (C) for the Block and Daily Undulating Groups.

Measurement Time	Testosterone (nmols × L^−1^)						Cortisol (nmols × L^−1^)						T:C Ratio					
	TRA			DUP			TRA			DUP			TRA			DUP		
	Mean	±	SD	Mean	±	SD	Mean	±	SD	Mean	±	SD	Mean	±	SD	Mean	±	SD
T1	18.4	±	6.1	20.9	±	7.6	428.1	±	102.1	410.6	±	70.4	4.7	±	2.5	5.4	±	2.6
T2	19.9	±	6.7	20.5	±	10.1	404.3	±	149.9	425.7	±	73.7	5.9	±	4.1	4.8	±	1.9
T3	19.2	±	7.3	23.5	±	7.0	428.6	±	142.5	474.9	±	70.2	4.6	±	1.5	5.0	±	1.7
T4	18.3	±	6.6	22.5	±	6.9	345.4	±	183.4	383.6	±	177.2	8.0	±	1.0	7.5	±	5.0

Note: T1 = pre-testing, T2 = after 4 weeks of training, T3 = after 8 weeks of training, and T3 = after 11 weeks of training. DUP = daily undulating, TRA = traditional training.

**Table 2:** Relationship of T, C, and T:C with % gain in isometric peak force (IPF) and scaled isometric peak force (IPFa).

Group	Initial T	Final T	Initial C	Final C	Initial T:C	Final T:C
Combined ΔIPF_T1-T4_	0.31	0.28	−0.53 *	−0.23	0.64 *	0.79 *
Block ΔIPF_T1-T4_	0.31	0.27	−0.09	−0.48	0.69 *	0.86 *
DUP ΔIPF_T1-T4_	0.31	0.31	−0.74 *	−0.16	0.21	0.66 *
Combined ΔIPFa_T1-T4_	0.31	0.23	−0.56 *	−0.31	0.62 *	0.77 *
Block ΔIPFa_T1-T4_	0.27	0.27	−0.12	−0.51	0.61 *	0.84 *
DUP ΔIPFa_T1-T4_	0.18	0.26	−0.77 *	−0.22	0.2	0.60

* Statistically significant Pearson’s correlation coefficient (*p* < 0.05); Initial = T_1_, Final = T_4_.
